# Clinical and economic impact of partnered pharmacist medication charting in the emergency department

**DOI:** 10.3389/fphar.2023.1273657

**Published:** 2023-12-08

**Authors:** Tesfay Mehari Atey, Gregory M. Peterson, Mohammed S. Salahudeen, Tom Simpson, Camille M. Boland, Ed Anderson, Barbara C. Wimmer

**Affiliations:** ^1^ School of Pharmacy and Pharmacology, College of Health and Medicine, University of Tasmania, Hobart, TAS, Australia; ^2^ Pharmacy Department, Royal Hobart Hospital, Tasmanian Health Service, Hobart, TAS, Australia

**Keywords:** partnered pharmacist, co-charting, medication charting, emergency department, length of stay, cost-benefit, cost-effectiveness, PPMC

## Abstract

**Introduction:** Partnered pharmacist medication charting (PPMC), a process redesign hypothesised to improve medication safety and interdisciplinary collaboration, was trialed in a tertiary hospital’s emergency department (ED).

**Objective:** To evaluate the health-related impact and economic benefit of PPMC.

**Methods:** A pragmatic, controlled study compared PPMC to usual care in the ED. PPMC included a pharmacist-documented best-possible medication history (BPMH), followed by a clinical conversation between a pharmacist and a medical officer to jointly develop a treatment plan and chart medications. Usual care included medical officer-led traditional medication charting in the ED, without a pharmacist-obtained BPMH or clinical conversation. Outcome measures, assessed after propensity score matching, were length of hospital or ED stay, relative stay index (RSI), in-hospital mortality, 30-day hospital readmissions or ED revisits, and cost.

**Results:** A total of 309 matched pairs were analysed. The median RSI was reduced by 15.4% with PPMC (*p* = 0.029). There were no significant differences between the groups in the median length of ED stay (8 vs*.* 10 h, *p* = 0.52), in-hospital mortality (1.3% vs*.* 1.3%, *p* > 0.99), 30-day readmission rates (21% vs*.* 17%; *p* = 0.35) and 30-day ED revisit rates (21% vs*.* 19%; *p* = 0.68). The hospital spent approximately $138.4 for the cost of PPMC care per patient to avert at least one medication error bearing high/extreme risk. PPMC saved approximately $1269 on the average cost of each admission.

**Conclusion:** Implementing the ED-based PPMC model was associated with a significantly reduced RSI and admission costs, but did not affect clinical outcomes, noting that there was an additional focus on medication reconciliation in the usual care group relative to current practice at our study site.

## 1 Introduction

Interventions that involve the integration of pharmacists in the emergency department (ED) have been associated with significant reductions in polypharmacy, medication errors and the use of potentially inappropriate medications (Becerra-Camargo et al., 2013; Koehl et al., 2019; Parro Martín et al., 2021; Atey et al., 2022). However, whether these benefits translate into health and economic outcomes is not clear in the literature. Conflicting evidence exists regarding the impact of ED-based pharmacist interventions on these outcomes. Some studies reported that no significant benefit was gained from the interventions on the length of hospital stay (LOS) (DeClifford et al., 2007; DeLorenzo-Pinto et al., 2018; Pevnick et al., 2018), length of ED stay (Mortimer et al., 2011; Robey-Gavin and Abuakar, 2016), in-hospital mortality (DeLorenzo-Pinto et al., 2018; Kulwicki et al., 2019; Kozlow and Livings, 2021), and hospital readmissions (DeLorenzo-Pinto et al., 2018; Pevnick et al., 2018) and ED revisits (DeLorenzo-Pinto et al., 2018) at 30 days after discharge. By contrast, others reported significant positive effects on the LOS (Masic et al., 2019; Kozlow and Livings, 2021) and length of ED stay (Masic et al., 2019). Evidence from interventions delivered by pharmacists conducted in other hospital settings also remains heterogeneous, with the majority reporting inconclusive impact on mortality and rates of readmissions and ED revisits at 3, 6, and 12 months (Thomas et al., 2014; Christensen and Lundh, 2016; Ravn-Nielsen et al., 2018; Santolaya-Perrin et al., 2019).

An example of an ED process redesign is partnered pharmacist medication charting (PPMC), a relatively new initiative intended to improve medication safety. With PPMC, a pharmacist obtains the best-possible medication history (BPMH) for the patient and has a clinical conversation with a medical officer to jointly develop a treatment plan and chart medications. Previously published studies have largely focused on the impact of PPMC on medication errors, LOS and cost of admission in older patients taking complex medications (Tong et al., 2015; Department of Health and Human Services, 2018). There remains a paucity of evidence on the impact of PPMC on health-related and economic outcomes in patients presenting to ED. We hypothesised that health- and economic-related benefits of PPMC may arise from a decreased incidence of medication errors potentially bearing high/extreme risks and from having a clear initial treatment plan in place. Therefore, we aimed to investigate the impact of PPMC on health- and economic-related outcomes compared to usual care, specifically, on the LOS, relative stay index (RSI, which is a risk-adjusted LOS) (Australian Institute of Health and Welfare, 2022a), length of ED stay, in-hospital mortality, and 30-day hospital readmissions and ED revisits. The cost-effectiveness and cost-benefit of PPMC were also determined.

## 2 Materials and methods

A detailed account of the study setting, study population, study period, study design, study arms, inclusion and exclusion criteria, and data collection procedures are presented elsewhere (Atey et al., 2023). A pragmatic, parallel controlled study was conducted with a study population composed of adults (aged 18 years or older) visiting the ED and taking at least one medication. This study included patients subsequently admitted to a general medicine unit (GMU) or emergency medicine unit (EMU) between 1 June 2020, and 17 May 2021.

In the PPMC arm, a best-possible medication history (BPMH) was promptly documented by an ED pharmacist at the earliest possible time point in the ED. The BPMH was obtained through structured patient interviews and secondary sources, such as caregivers, electronic health records, and community pharmacies. Following clinical review, the pharmacist collaborated with a medical officer (at least a post-graduate year 2 resident) to develop a shared medication treatment plan (SMTP). The co-signed SMTP was placed in the patient’s medical progress notes as a record of the shared clinical decision-making. Based on the SMTP, medications were then charted by the pharmacist using purple ballpoint ink. The medical officer endorsed each medication order before administration by nursing staff. A ward pharmacist later conducted a medication reconciliation (MedRec) on the inpatient ward.

**FIGURE 1:**
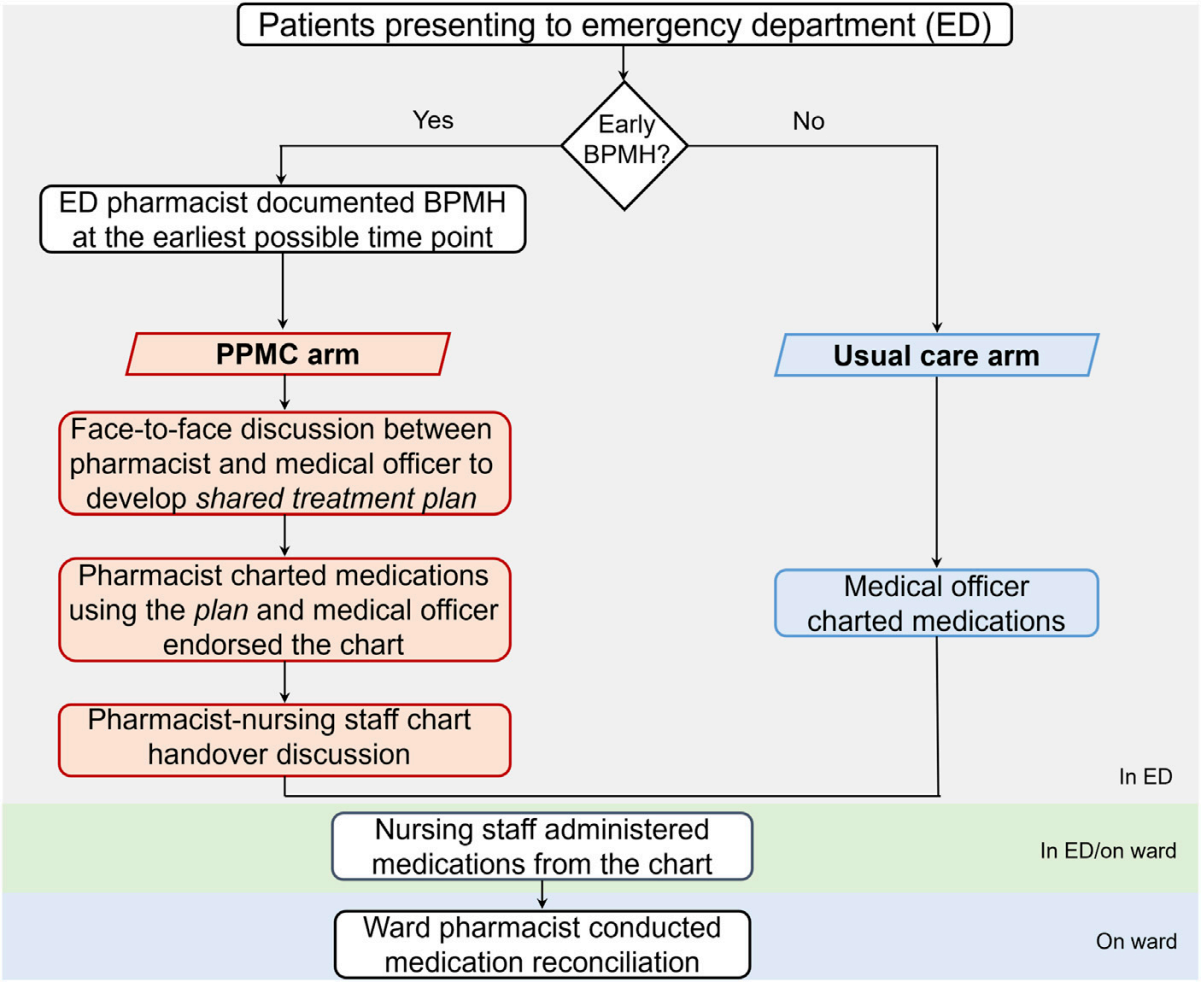
Admission processes for patients in the PPMC and usual care arms. Abbreviations: BPMH, best-possible medication history; ED, emergency department; PPMC, partnered pharmacist medication charting.

The usual care arm included patients who followed the standard admission process. This process entailed the traditional medication charting, wherein a medical officer wrote medication charts within the ED using black/blue ballpoint ink. Notably, the charting occurred in the absence of a pharmacist-collected BPMH or any pharmacist-medical officer collaborative discussion in the ED. Following this, a ward pharmacist conducted a MedRec on the inpatient ward (Figure 1).

### 2.1 Independent variables

Independent socio-demographic and clinical variables were age (in years), sex (male or female), age-adjusted Charlson comorbidity index (CCI), Australasian Triage Scale, type of admission specialty units (GMU or EMU), the arrival time of the day [during peak business hours (9:00–15:00) or outside these hours (8:00–9:00 or 15:00–21:00)] and the arrival day of the week (weekdays or weekends/public holidays). A triage scale is a clinical tool consisting of 5 ED presentation levels, with 1 being the most critical and 5 being the least critical (Australasian College for Emergency Medicine, 2000). The CCI encompasses 17 medical conditions and a patient’s age, with total scores ranging from 0 to 33 (Charlson et al., 1987).

### 2.2 Outcome measures

Primary outcomes were the LOS from the time of the ED presentation to the cessation of the acute episode of care (discharge), in days, and the RSI. The RSI was computed by dividing a patient’s actual LOS by an expected LOS for a similar patient, which was based on national figures (Independent Health and Aged Care Pricing Authority, 2021). The expected LOS is standardised across Australian-refined diagnosis-related groups (AR-DRGs), care type, age, admission type, arrival source, discharge destination and comorbidity level (i.e., number of comorbidities from separate International Classification of Diseases-10 Australian Modification (ICD-10-AM) chapters) (Australian Institute of Health and Welfare, 2022a). An RSI greater than one indicates that a patient’s LOS is longer than would be expected, given the patient’s casemix distribution. An AR-DRG is “an Australian admitted patient classification system which provides a clinically meaningful way of relating the number and type of patients treated in a hospital (known as hospital casemix) to the resources required by the hospital” (Australian Institute of Health and Welfare, 2022b). Each AR-DRG has an alphanumeric code that represents a group of patients with similar clinical conditions demanding similar hospital services.

The expected LOS data were obtained from the Independent Health and Aged Care Pricing Authority’s National Hospital Cost Data Collection (NHCDC) report (version 10.0) (Independent Health and Aged Care Pricing Authority, 2021). The NHCDC public sector’s 23rd round report for the fiscal year 2018-19 was the latest publicly available at the time of manuscript writing. The report also contains hospital costs of admission data by jurisdictions and the episode of admission type (e.g., acute, subacute or non-acute admissions). The NHCDC statistics were linked to each patient’s data using AR-DRG as a linking variable.

Secondary outcomes included length of ED stay from ED presentation to ED departure (in hours), all-cause in-hospital mortality during the acute admission, occurrences of unplanned hospital readmissions or ED revisits to the same hospital within 30 days after hospital discharge, and PPMC’s cost-effectiveness and cost-benefit. The economic analysis was conducted from a health system’s perspective. The benefits from cost avoidance with PPMC based on the potential to avert a high/extreme risk error (Atey et al., 2023) (cost-effectiveness) and from cost savings by reducing the RSI (cost-benefit) were estimated, as described below. The cost of PPMC was estimated relative to the cost of usual care.

Firstly, the cost of care per patient to avert at least one high/extreme risk was estimated based on a) the average time required to deliver PPMC for one patient, b) the number of patients who needs to be treated (NNT) with PPMC to prevent at least one high/extreme risk and c) the hourly salary rate. The average time estimate to conduct one PPMC activity was obtained from the Royal Hobart Hospital (RHH), in which an online survey of PPMC pharmacists was conducted and then a consensus was reached among the pharmacists. The NNT was based on our PPMC’s impact on medication error study, and definitions and prevalence of high or extreme-risk errors are provided elsewhere (Atey et al., 2023). The estimated hourly salary rates were based on data from THS’s 2020/21 Public Sector Agreements (Tasmanian Health Service, 2019); $46.3 for a PPMC pharmacist based on Allied Health Professional Level 2 Year 4 (Department of Health and Human Services, 2018), $55.8 for a supervising pharmacist based on Allied Health Professional Level 4 Year 2, $54.0 for an ED medical officer based on Medical Practitioner Level 7 (Registrar Year 3), and $45.0 for a nurse based on Registered Nurse Grade 4 Year 1.

Secondly, the relative reduction of RSI with PPMC was translated into admission cost savings by multiplying the percentage of RSI reduction by the average cost of unplanned, acute admission in Tasmania. The cost of admission in Tasmania was based on the NHCDC report (Independent Health and Aged Care Pricing Authority, 2021). According to the report’s historical cost trend from 2016 to 17 to 2018-19, the average cost of admission increased by 6.6% annually in Tasmania. We factored in this increase for estimating the cost of admission for the study period (2020-21). Based on the national data, the average “expected LOS” trend remained relatively steady; therefore, no adjustments were considered in this study.

Thirdly, the cost difference between admission cost savings and the cost of care was reported as the PPMC cost-benefit. Approximate fixed costs from the cost of initial credentialling were also estimated; however, we were unable to incorporate some of PPMC’s fixed costs, such as the cost of establishment and supervision due to a lack of data. All the cost findings presented in this study were only approximations and were reported in Australian dollars.

### 2.3 Propensity score matching

A 1:1 nearest neighbour propensity score matching without replacement using a calliper of 0.03 was used to create comparable groups in both arms. The propensity scores were calculated using a logistic regression based on the covariates (CCI, arrival time of the day and admission unit type). The quality of matching was assessed using numerical summaries and graphical methods. A covariate balance before and after matching was deemed adequate when a standardised mean difference was below 0.1 (Harder et al., 2010). A MatchIT package was used for the propensity score matching (Stuart et al., 2011).

### 2.4 Statistical analysis

The normality of data distribution was checked via statistical methods using the Shapiro-Wilk test and visual inspection of histograms and Q-Q plots. Multicollinearity was determined by observing the size of correlation coefficients, variance inflation factors and eigenvalues. Prior to matching, statistics were compared using the Wilcoxon rank-sum test for continuous data and Pearson’s chi-square test for categorical data. For the matched pairs, continuous variables were compared using the Wilcoxon signed-rank test, and categorical variables were compared using McNemar’s chi-square test (Swinscow and Campbell, 1997; Austin, 2008).

Subgroup analysis according to the major diagnostic category (MDC) was conducted to examine whether the RSI varied by MDC between the two study groups. The MDCs are mutually exclusive categories into which all possible DRGs and principal diagnoses fall (Independent Health and Aged Care Pricing Authority, 2020). The diagnosis in each MDC corresponds to a single aetiology or body system, for example, “MDC 01 Diseases and Disorders of the Nervous System.” The analysis was compared using the Wilcoxon rank sum test.

Kaplan-Meier’s “survival” probability curves were constructed to analyse time to hospital readmissions and ED revisits. Patients who died in the hospital and their counter-matched pairs were excluded from health-related outcome analyses. All reported *p*-values <0.05 were considered statistically significant. Data were analysed in R^®^ version 4.1.12 (R Foundation for Statistical Computing, Vienna, Austria) (R Core Team, 2013).

## 3 Results

### 3.1 Patients’ characteristics

**FIGURE 2:**
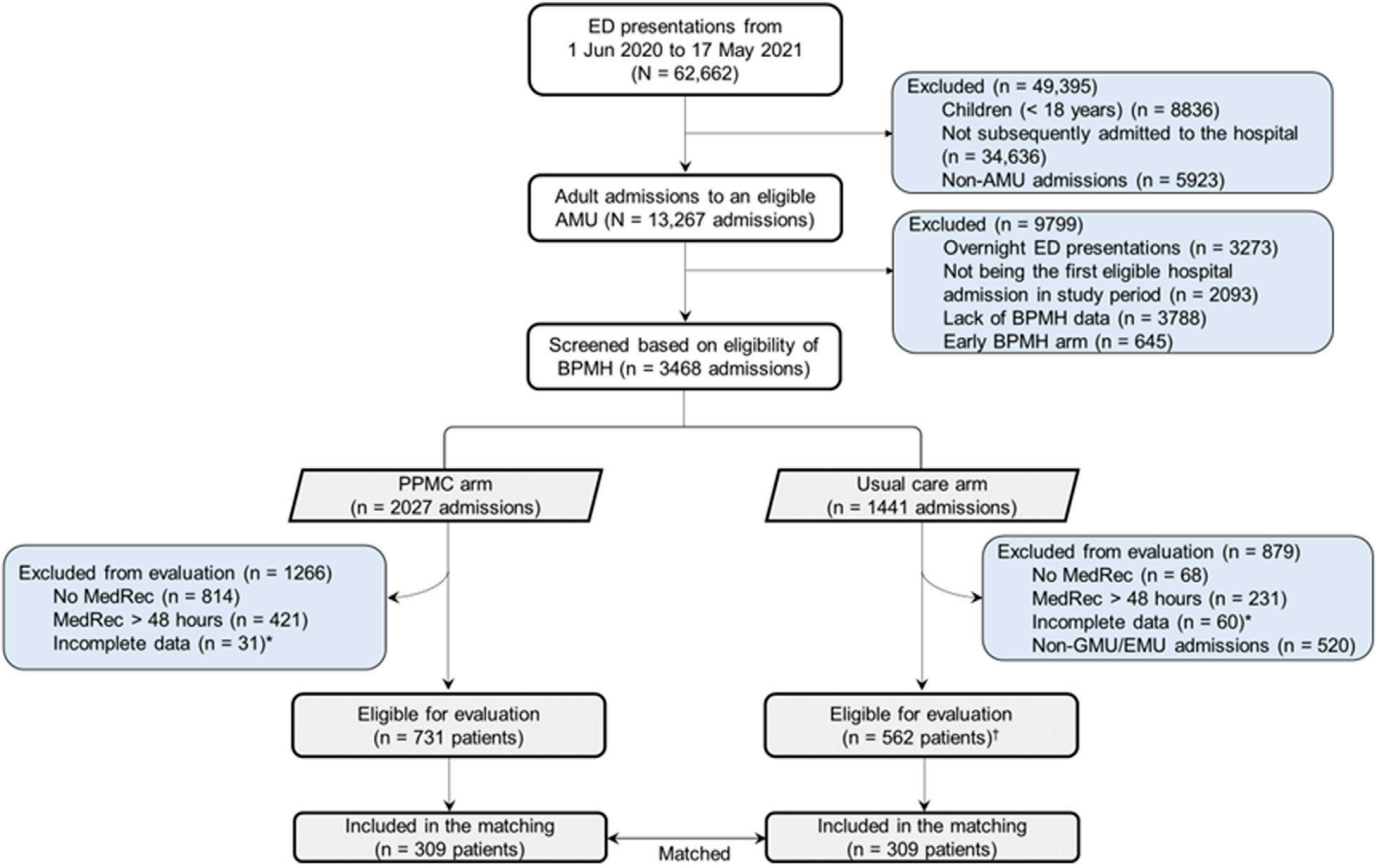
Screening and selection of the participants. Abbreviations: AMU, acute medical unit; BPMH, best-possible medication history; ED, emergency department; EMU, emergency medicine unit; GMU, general medicine unit; MedRec, medication reconciliation; PPMC, partnered pharmacist medication charting *Examples include incomplete/unavailable diagnosis-related group, discharge summary or medication chart information.

The screening and selection of the study participants are detailed elsewhere (Atey et al., 2023). There were 3,468 admissions to GMU and EMU screened based on the eligibility of BPMH in both cohorts during the study period (Figure 2). Of these, 731 and 562 eligible patients made up the PPMC arm and the usual care arm, respectively. A total of 309 patients from the usual care group were matched to the same number of patients from the PPMC group.

The demographic and clinical characteristics of each cohort before and after matching are tabulated in Table 1. Sex, triage level and arrival day of the week were comparable between the groups before matching. However, there were significant differences (*p* < 0.001) in the median age, CCI, types of admission units and arrival time of the day between the unmatched groups. The baseline characteristics of the cohorts were well-balanced post-matching, with a standardised mean difference of less than 0.1. The median ages were 77 years [interquartile range (IQR): 64–86 years] and 75 years (IQR: 63–84 years) in the matched PPMC group and the usual care group, respectively.

**TABLE 1:** Unmatched and matched comparisons between the PPMC group and the usual care group.

Variables	Unmatched comparison				Matched comparison			
	PPMC (*N* = 731)	Usual care (*N* = 562)	*p*-value	SMD	PPMC (*N* = 309)	Usual care (*N* = 309)	*p*-value	SMD
Sex female, n (%)	402 (55%)	288 (51%)	0.18^a^		162 (52%)	166 (54%)	0.74^a^	
Median age in years (IQR)	79 (69, 86)	75 (63, 84)	**< 0.001** ^b^		77 (64, 86)	75 (63, 84)	0.36^b^	
Charlson comorbidity index	5 (4, 6)	5 (3, 6)	**< 0.001** ^b^	0.2342	5 (3, 6)	5 (3, 6)	0.84^b^	0.018
Australasian triage scale, n (%)	3 (2, 4)	3 (3, 4)	0.67^b^		3 (3, 4)	3 (3, 3)	0.64^b^	
Admission unit			**< 0.001** ^a^				>0.99^b^	
Emergency medicine	79 (11%)	192 (34%)		0.7523	78 (25%)	79 (26%)		0.0104
General medicine	652 (89%)	370 (66%)		−0.7523	231 (75%)	230 (74%)		−0.0104
Arrival time of the day			**< 0.001^a^**				>0.99^b^	
Within business hours (9 a.m.–5 p.m.)	655 (90%)	282 (50%)		1.2917	233 (75%)	234 (76%)		−0.0106
Outside business hours	76 (10%)	280 (50%)		−1.2917	76 (25%)	75 (24%)		0.0106
Arrival day of the week			0.37^a^				0.58^b^	
Workdays (Monday to Friday)	453 (62%)	362 (64%)			178 (58%)	185 (60%)		
Weekends including public holidays	278 (38%)	200 (36%)			131 (42%)	124 (40%)		

Abbreviations: IQR, interquartile range; n, number; PPMC, partnered pharmacist medication charting; SMD, standardised mean difference.

^a^ Pearson’s chi-square test for unmatched comparison; McNemar’s chi-square test for matched comparison.

^b^ Wilcoxon rank sum test for unmatched comparison; Wilcoxon signed rank test for matched comparison.

Bold highlights values that have statistical significance.

### 3.2 Length of hospital stay and relative stay index

The median absolute LOS did not vary significantly between the groups (*p* = 0.51), with 3.9 days (IQR: 2.1, 7.2) in the PPMC group and 4.2 days (IQR: 2.6, 7.9) in the usual care group (Table 2). In contrast, patients in the PPMC group had a lower RSI (1.1; IQR: 0.7, 1.9) than those in the usual care group (1.3; IQR, 0.8, 2.1) (*p* = 0.029). The RSI was reduced by 15.4% with PPMC compared to the usual care group.

**TABLE 2:** Impact of PPMC on health-related outcomes and cost of admission.

Outcomes, median (IQR) or *n* (%)	PPMC (N = 301)	Usual care (N = 301)	*p*-value
Length of hospital stay in days	3.9 (2.1, 7.2)	4.2 (2.6, 7.9)	0.51^a^
Relative stay index	1.1 (0.7, 1.9)	1.3 (0.8, 2.1)	**0.029** ^a^
Length of ED stay in hours	8 (5, 14)	10 (7, 13)	0.52^a^
30-day hospital readmissions	63 (21%)	52 (17%)	0.35^b^
30-day ED revisits	63 (21%)	57 (19%)	0.68^b^

Abbreviations: ED, emergency department; IQR, interquartile range; n, number; PPMC, partnered pharmacist medication charting.

^a^ Wilcoxon signed rank test.

^b^ McNemar’s chi-square test.

Bold highlights values that have statistical significance.

**FIGURE 3:**
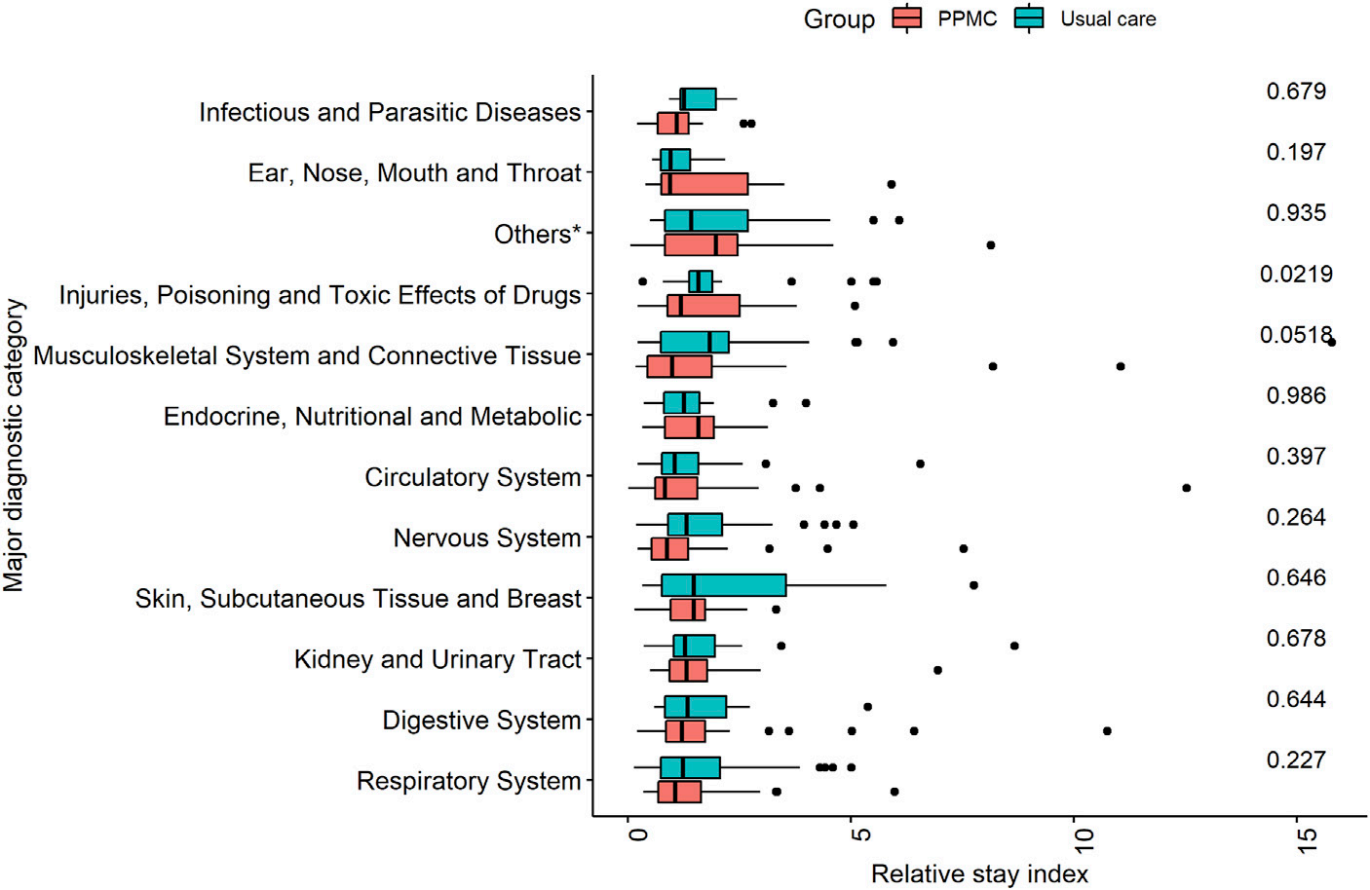
Comparison of relative stay index by major diagnostic category in the PPMC group and the usual care group. *Others: Alcohol/Drug Use, Hepatobiliary System and Pancreas, Mental Diseases and Disorders, Blood and Blood Forming Organs and Immunological Disorders, and Diseases and Disorders of Ear, Nose, Mouth and Throat.

After conducting a sub-group analysis based on MDC, the only statistically significant difference (*p* = 0.022) in the median RSI was for the principal diagnosis related to “*Injuries, Poisoning and Toxic Effects of Drugs*.” Within this diagnostic category, patients in the PPMC group exhibited a comparatively shorter median RSI (1.2; IQR: 0.89, 2.50) than those in the usual care group (1.58; IQR: 1.37, 1.90) (Figure 3).

### 3.3 Other health-related outcomes

There were no statistically significant differences between the two groups for the median length of ED stay (Table 2). All-cause in-hospital mortality occurred in 4 patients (1.3%) in each of the groups (*p* > 0.99). The majority of the patients (∼80%) in both groups were discharged to their usual home residence following the cessation of the acute episode of care.

**FIGURE 4:**
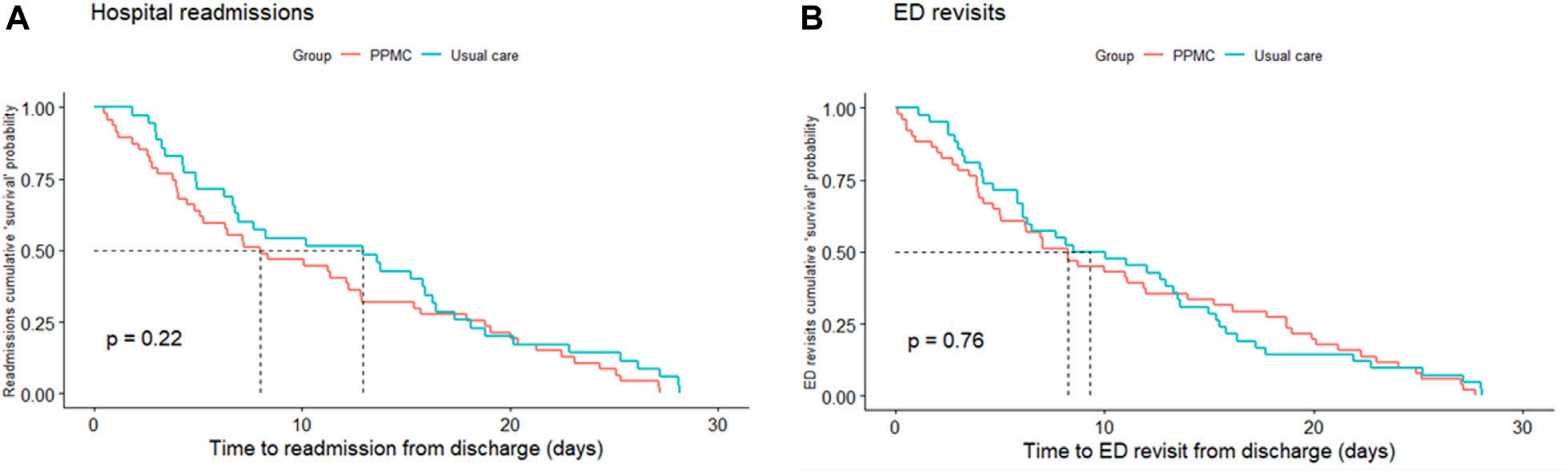
Kaplan-Meier cumulative “survival” probability curves for hospital readmissions **(A)** and ED revisits **(B)** within 30 days after hospital discharge. Abbreviations: ED, emergency department; PPMC, partnered pharmacist medication charting.

Figure 4 shows Kaplan-Meier “survival” curves for the hospital readmissions and ED revisits within 30 days after the hospital discharge. Time-to-event analyses did not show any significant between-group differences in the time to hospital readmissions (*p* = 0.22) or ED revisits (*p* = 0.76).

### 3.4 Cost-effectiveness and cost-benefit of PPMC

The fixed cost of initial PPMC credentialling was estimated at $330.2 per pharmacist (Table 3). The average time required to deliver PPMC for a single patient was estimated to be 75 min, with the most time (73.3%, 55 min) spent on determining the BPMH (i.e., 35 min) and charting medications (i.e., 20 min). The hospital spent approximately $138.4 for the cost of PPMC care per patient to avert at least one medication error bearing high/extreme risk (Table 4).

**TABLE 3:** Fixed cost of initial PPMC credentialling.

Cost of initial credentialling	Personnel	Estimated time spent in hours	Salary rate per hour	Number of trainers	Total	Comment
Activities						
Six PPMC clinical episodes	Hospital pharmacists	3.5 h^a^	$46.3 per h	-	$162.0	AHP Level 2 Year 4
Supervised clinical case studies	Supervising pharmacists	0.875 h^b^	$55.8 per h	2	$48.8	AHP Level 4 Year 2
OSCE	Supervising pharmacist	1 h	$55.8 per h	1	$55.8	AHP Level 4 Year 2
	Medical officer	1 h	$63.7 per h	1	$63.6	Medical Practitioner Level 10
				Total	**$330.2**	Per pharmacist

Abbreviations: AHP, allied health professional; h, hours; OSCE, objective structured clinical exam.

^a^ 30–40 min per one clinical episode (average 35 min): 35 min * 6 episodes = 3.5 h.

^b^ Supervision of clinical episodes was conducted in batches in a rolling fashion: i.e., 6 clinical episodes for 4 pharmacists: 3.5 h/4 pharmacists = 0.875 h per pharmacist.

**TABLE 4:** Breakdown of the cost analysis according to the approximate input cost parameters.

**A. Cost of PPMC care**	**Estimated time spent per patient**	**Estimated cost**	**Comment**
**PPMC activities** ^a^			
Obtaining a BPMH	35 min	-	-
Holding a clinical conversation with a medical officer and writing a shared treatment plan	10 min	-	-
Writing a medication chart	20 min	-	-
Nursing handover discussion	5 min	-	-
Other PPMC activities such as triaging/screening	5 min	-	-
**Total time spent in PPMC^b^ ^,^ ^c^**			
By a pharmacist	10 + 20 + 5 + 5 = 40 min → 0.67 h	0.67 h ✕ $46.3 per h = $31.02	AHP Level 2 Year 4
By a nursing staff	5 min → 0.08 h	0.08 h ✕ $45.00 per h = $3.60	Registered Nurse Grade 4 Year 1
**Total cost per patient**		**$34.62**	$34.62 = $31.02 + $3.60

**B. PPMC cost-benefit**	**Cost**		**Comment**
Cost per patient	$34.62		
Cost of PPMC care to prevent one high/extreme risk	$138.4		NNT^d^: 4 ✕ $34.62
Cost saving using RSI	$1,303.6		15.4% of $8,465.6^e^
Cost-difference	$1,269		$1,303.6 - $34.62

Abbreviations: AHP, allied health professional; h, hours; min, minutes; NNT, number of patients who need to be treated; RSI, relative stay index.

^a^ These time points were estimated based on an average number of pre-admission medicines (including regular, PRN, and complementary medicines), i.e., ten medicines for one PPMC, patient (Atey et al., 2023).

^b^ As the BPMH was a common element in both arms, it was deemed cost-neutral and therefore not included in the analysis.

^c^ The time spent by medical officers on clinical discussions was not included in the analysis because their involvement in these discussions did not require any extra time compared to usual care.

^d^ The NNT with PPMC to prevent at least one high/extreme risk error compared to usual care was obtained from the medication discrepancy/error study (Atey et al., 2023).

^e^ The RSI, relative risk reduction was 15.4% with PPMC, compared to the usual care group. The average cost of admission in Tasmania in 2020–21 for unplanned, acute admissions was $8,465.6, according to the projected NHCDC data (Independent Health and Aged Care Pricing Authority, 2021).

The average cost of an unplanned, acute admission in Tasmania was estimated to be $8,465.6 in 2020-21, based on an average 6.6% annual increase in the trend of admission costs (Supplementary Appendix) (Independent Health and Aged Care Pricing Authority, 2021). With PPMC, a notable reduction of 15.4% in the RSI was achieved, translating into proportional reductions in the cost of admission. This translated to an approximate saving of $1,303.6 per admission per patient. After accounting for the cost of PPMC care at $34.6 per patient, it can be estimated that PPMC would yield net savings of approximately $1,269 per admission per patient.

## 4 Discussion

Compared to the trial’s usual care, the RSI was significantly reduced by 15.4% with PPMC. However, there were no statistically significant between-group differences in the other outcomes. The hospital spent approximately $138.4 per patient on PPMC to prevent at least one high/extreme risk medication error. Approximately $1,269 was also saved per admission per patient with PPMC. A previous study reported each admission under the PPMC arm had an average saving of $726, probably from increased safety and reduced complications (Department of Health and Human Services, 2018). The previous study was conducted in 2016-17, while our study period covered 2020-21, leaving a possibility of cost variations.

Previous studies have shown that patients’ LOS may be prolonged due to serious/severe preventable adverse drug events (Bates et al., 1997; Pinilla et al., 2006). High/extreme risk errors could result in temporary or permanent injury and necessitate additional monitoring or intervention, thereby prolonging hospitalisation. The implementation of PPMC reduced the occurrence of these high/extreme risk errors (Atey et al., 2023), and fostered collaboration between a pharmacist and a medical officer through the development of a clear, comprehensive patient treatment plan. This plan may facilitate timely investigations and improve communication among clinicians, thus streamlining the discharge process and affecting the RSI. Conversely, medication inaccuracies and inadequately planned plans may hinder timely investigations (Gleason et al., 2010; Tong et al., 2020), potentially affecting the duration of stay.

Reduction of the RSI and cost of admission, and potential time savings with PPMC could have enormous clinical and financial implications for hospitals with high bed occupancy rates or those suffering from significant access blocks. In September 2020, more than two-thirds of patients waiting for hospital admissions across 93 Australian EDs were experiencing access blocks (Australasian College for Emergency Medicine, 2018). Access block accounts for an increased ED staff workload and a prolonged LOS, and it may delay the initiation of treatments, creating a negative feedback loop for patient care (Richardson, 2002; Bernstein et al., 2009; Kennebeck et al., 2011; Australasian College for Emergency Medicine, 2018; Morley et al., 2018). Minimising any delay in hospital flow is, therefore, clinically and financially important for hospitals marked by bed shortages and overcrowding.

The findings from the sub-group analysis of patients with a principal diagnosis related to “*Injuries, Poisoning, and Toxic Effects of Drugs*” shed light on the potential benefits of PPMC in specific clinical contexts. These patients may have medication misadventure associated with confusion or poor communication about their drug therapy. PPMC can streamline medication charting, improve communication among clinicians and facilitate quicker access to vital medication information, thus potentially impacting medication-related processes and improving patient outcomes. While this sub-group analysis provided valuable preliminary insights, it is important to acknowledge that its robustness was limited by the modest size of the matched sample. Therefore, caution should be taken when interpreting the findings, and future studies should prioritise replicating this sub-group analysis using a larger and more diverse sample.

This study has several strengths and limitations to consider. We used a robust propensity-matched analysis endeavouring to create cohorts from both groups that were comparable in baseline characteristics. This approach is currently recommended as a standard tool for investigators trying to estimate the effects of interventions in non-randomised studies (Austin, 2011). A comprehensive panel evaluation methodology was employed for the assessment of medication errors.

Due to ethical concerns about denying patients access to a new service, randomisation of the patients was not possible. Although we used matching techniques, the absence of randomisation still makes it impossible to rule out the possibility of other potentially unknown or residual confounders. The unavailability of a patient’s actual cost of admission data is another limitation, which is a potential area for future study. Our analysis was limited to easily quantifiable, short-term outcomes (e.g., RSI) and did not include additional variables that may have longer-term or more difficult-to-measure consequences (e.g., quality of life).

It is important to note that “usual care” in this trial was operationally defined as patients receiving MedRec (including BPMH) on the inpatient ward within 48 h of admission. This definition was used for the purpose of assessing medication errors, mimicking ideal practice, and ensuring comparability between the study groups (Atey et al., 2023). However, it is worth noting that in practice, MedRec is not universally conducted for all admitted patients at our study hospital. In fact, only about one-third of patients currently receive this on the wards. This means that the true impact of PPMC, if implemented within the current environment, might have been underestimated in our study.

The analysis omitted the time spent by medical officers during their involvement in the PPMC’s clinical discussions. This decision was based on the understanding that their participation did not demand additional time compared to the usual care scenario, where they independently obtained medication histories and charted medications. In fact, it was anecdotally reported that their time commitment had been reduced with PPMC. For future studies, however, a rigorous time and motion study is recommended to accurately estimate the time spent on each activity.

The study used secondary clinical and administrative data collected from routine hospital patient care, potentially affecting the completeness and accuracy of data. Being a single-site study may limit the study’s generalisability to other populations or settings. Therefore, it is important to consider conducting a multi-site study in the future and extending PPMC to cover additional clinical areas and/or extended working hours.

## 5 Conclusion

PPMC within the ED was associated with a significantly reduced relative stay index and has the potential to reduce costs per admission. However, no statistically significant differences were observed between PPMC and usual care in the ED stay, in-hospital mortality, and 30-day hospital readmissions or ED revisits, noting that there was an additional focus on medication reconciliation in the usual care group relative to current practice at our study site.

## Data Availability

The datasets presented in this article are not readily available because “only individuals named in the ethics application are authorised to access the data”. Requests to access the datasets should be directed to MS at Mohammed.Salahudeen@utas.edu.au.

## References

[B1] Atey T. M. Peterson G. M. Salahudeen M. S. Bereznicki L. R. Simpson T. Boland C. M. (2023). Impact of partnered pharmacist medication charting (PPMC) on medication discrepancies and errors: a pragmatic evaluation of an emergency department-based process redesign. Int. J. Environ. Res. Public Health 20, 1452. 10.3390/ijerph20021452 36674208 PMC9859430

[B2] Atey T. M. Peterson G. M. Salahudeen M. S. Bereznicki L. R. Wimmer B. C. (2022). Impact of pharmacist interventions provided in the emergency department on quality use of medicines: a systematic review and meta-analysis. Emerg. Med. J. 40, 120–127. 10.1136/emermed-2021-211660 35914923

[B3] Austin P. C. (2008). A critical appraisal of propensity-score matching in the medical literature between 1996 and 2003. Stat. Med. 27, 2037–2049. 10.1002/sim.3150 18038446

[B4] Austin P. C. (2011). An introduction to propensity score methods for reducing the effects of confounding in observational studies. Multivar. Behav. Res. 46, 399–424. 10.1080/00273171.2011.568786 PMC3144483 21818162

[B5] Australasian College for Emergency Medicine (2000). Policy on the australasian triage scale. ACEM. Available at: https://acem.org.au/Content-Sources/Advancing-Emergency-Medicine/Better-Outcomes-for-Patients/Triage (Accessed May 5, 2021).

[B6] Australasian College for Emergency Medicine (2018). The long wait: an analysis of mental health presentations to Australian emergency departments. Available at: https://acem.org.au/getmedia/60763b10-1bf5-4fbc-a7e2-9fd58620d2cf/ACEM_report_41018 (Accessed February 2, 2022).

[B7] Australian Institute of Health and Welfare (2022a). Hospitals info & downloads: relative stay index (RSI) . Available at: https://www.aihw.gov.au/reports-data/myhospitals/content/glossary (Accessed September 17, 2022).

[B8] Australian Institute of Health and Welfare (2022b). Australian refined diagnosis-related groups (AR-DRG). AIHW. Available at: https://www.aihw.gov.au/reports/hospitals/ar-drg-data-cubes/contents/about (Accessed September 20, 2022).

[B9] Bates D. W. Spell N. Cullen D. J. Burdick E. Laird N. Petersen L. A. (1997). The costs of adverse drug events in hospitalized patients. Adverse Drug Events Prevention Study Group. JAMA 277, 307–311. 10.1001/jama.277.4.307 9002493

[B10] Becerra-Camargo J. Martinez-Martinez F. Garcia-Jimenez E. (2013). A multicentre, double-blind, randomised, controlled, parallel-group study of the effectiveness of a pharmacist-acquired medication history in an emergency department. BMC Health Serv. Res. 13, 337. 10.1186/1472-6963-13-337 23984830 PMC3844415

[B11] Bernstein S. L. Aronsky D. Duseja R. Epstein S. Handel D. Hwang U. (2009). The effect of emergency department crowding on clinically oriented outcomes. Acad. Emerg. Med. 16, 1–10. 10.1111/j.1553-2712.2008.00295.x 19007346

[B12] Charlson M. E. Pompei P. Ales K. L. Mackenzie C. R. (1987). A new method of classifying prognostic comorbidity in longitudinal studies: development and validation. J. Chronic Dis. 40, 373–383. 10.1016/0021-9681(87)90171-8 3558716

[B13] Christensen M. Lundh A. (2016). Medication review in hospitalised patients to reduce morbidity and mortality. Cochrane Database Syst. Rev. 20, CD008986. 10.1002/14651858.CD008986.pub3 PMC7119455 26895968

[B14] Declifford J. M. Caplygin F. M. Lam S. S. Leung B. K. (2007). Impact of an emergency department pharmacist on prescribing errors in an Australian Hospital. J. Pharm. Pract. 37, 284–286. 10.1002/j.2055-2335.2007.tb00766.x

[B15] Delorenzo-Pinto A. Garcia-Sanchez R. Herranz A. Miguens I. Sanjurjo-Saez M. (2018). Promoting clinical pharmacy services through advanced medication review in the emergency department. Eur. J. Hosp. Pharm. 27, 73–77. 10.1136/ejhpharm-2018-001599 32133132 PMC7043244

[B16] Department of Health and Human Services (2018). Health economic evaluation of the partnered pharmacist medication charting (PPMC) program. Melbourne, Victoria. Available at: https://www2.health.vic.gov.au/health-workforce/reform-and-innovation/partnered-pharmacist-medication-charting (Accessed March 5, 2020).

[B17] Gleason K. M. Mcdaniel M. R. Feinglass J. Baker D. W. Lindquist L. Liss D. (2010). Results of the Medications at Transitions and Clinical Handoffs (MATCH) study: an analysis of medication reconciliation errors and risk factors at hospital admission. J. Gen. Intern Med. 25, 441–447. 10.1007/s11606-010-1256-6 20180158 PMC2855002

[B18] Harder V. S. Stuart E. A. Anthony J. C. (2010). Propensity score techniques and the assessment of measured covariate balance to test causal associations in psychological research. Psychol. Methods 15, 234–249. 10.1037/a0019623 20822250 PMC2936698

[B19] Independent Health and Aged Care Pricing Authority (2021). National hospital cost data collection report, public sector, round 23 (financial year 2018–19). IHACPA. Available at: https://www.ihacpa.gov.au/health-care/costing/national-hospital-cost-data-collection (Accessed January 3, 2022).

[B20] Independent Health and Aged Care Pricing Authority (2020). Australian refined diagnosis related groups version 10.0. Available at: https://www.ihpa.gov.au/what-we-do/admitted-acute-care/ar-drg-version-10.

[B21] Kennebeck S. S. Timm N. L. Kurowski E. M. Byczkowski T. L. Reeves S. D. (2011). The association of emergency department crowding and time to antibiotics in febrile neonates. Acad. Emerg. Med. 18, 1380–1385. 10.1111/j.1553-2712.2011.01221.x 22168202

[B22] Koehl J. Steffenhagen A. Halfpap J. (2019). Implementation and impact of pharmacist-initiated home medication ordering in an emergency department observation unit. J. Pharm. Pract. 34, 459–464. 10.1177/0897190019879254 31594429

[B23] Kozlow E. A. Livings S. E. (2021). Time to four-factor prothrombin complex concentrate administration decreased by presence of a 24/7 pharmacist in the emergency department. J. Am. Coll. Clin. Pharm. 4, 33–39. 10.1002/jac5.1350

[B24] Kulwicki B. D. Brandt K. L. Wolf L. M. Weise A. J. Dumkow L. E. (2019). Impact of an emergency medicine pharmacist on empiric antibiotic prescribing for pneumonia and intra-abdominal infections. Am. J. Emerg. Med. 37, 839–844. 10.1016/j.ajem.2018.07.052 30097272

[B25] Masic D. Hidalgo D. C. Kuhrau S. Chaney W. Rech M. A. (2019). Pharmacist presence decreases time to prothrombin complex concentrate in emergency department patients with life-threatening bleeding and urgent procedures. J. Emerg. Med. 57, 620–628. 10.1016/j.jemermed.2019.06.027 31447188

[B26] Morley C. Unwin M. Peterson G. M. Stankovich J. Kinsman L. (2018). Emergency department crowding: a systematic review of causes, consequences and solutions. PLoS One 13, e0203316. 10.1371/journal.pone.0203316 30161242 PMC6117060

[B27] Mortimer C. Emmerton L. Lum E. (2011). The impact of an aged care pharmacist in a department of emergency medicine. J. Eval. Clin. Pract. 17, 478–485. 10.1111/j.1365-2753.2010.01454.x 21040247

[B28] Parro Martín M. D. L. Á. Muñoz García M. Delgado Silveira E. Martín‐Aragón Álvarez S. Bermejo Vicedo T. (2021). Intervention study for the reduction of medication errors in elderly trauma patients. J. Eval. Clin. Pract. 27, 160–166. 10.1111/jep.13407 32369877

[B29] Pevnick J. M. Nguyen C. Jackevicius C. A. Palmer K. A. Shane R. Cook-Wiens G. (2018). Improving admission medication reconciliation with pharmacists or pharmacy technicians in the emergency department: a randomised controlled trial. BMJ Qual. Saf. 27, 512–520. 10.1136/bmjqs-2017-006761 PMC5912995 28986515

[B30] Pinilla J. Murillo C. Carrasco G. Humet C. (2006). Case-control analysis of the financial cost of medication errors in hospitalized patients. Eur. J. Health Econ. 7, 66–71. 10.1007/s10198-005-0332-z 16369841

[B31] Ravn-Nielsen L. V. Duckert M.-L. Lund M. L. Henriksen J. P. Nielsen M. L. Eriksen C. S. (2018). Effect of an in-hospital multifaceted clinical pharmacist intervention on the risk of readmission: a randomized clinical trial. JAMA Intern 178, 375–382. 10.1001/jamainternmed.2017.8274 PMC5885912 29379953

[B32] R Core Team (2013). R: a language and environment for statistical computing. Vienna, Austria: R Foundation for Statistical Computing.

[B33] Richardson D. B. (2002). The access‐block effect: relationship between delay to reaching an inpatient bed and inpatient length of stay. Med. J. Aust. 177, 492–495. 10.5694/j.1326-5377.2002.tb04917.x 12405891

[B34] Robey-Gavin E. Abuakar L. (2016). Impact of clinical pharmacists on initiation of postintubation analgesia in the emergency department. J. Emerg. Med. 50, 308–314. 10.1016/j.jemermed.2015.07.029 26433427

[B35] Santolaya-Perrin R. Calderon-Hernanz B. Jimenez-Diaz G. Galan-Ramos N. Moreno-Carvajal M. T. Rodriguez-Camacho J. M. (2019). The efficacy of a medication review programme conducted in an emergency department. Int. J. Clin. Pharm. 41, 757–766. 10.1007/s11096-019-00836-0 31028596

[B36] Stuart E. A. King G. Imai K. Ho D. (2011). MatchIt: nonparametric preprocessing for parametric causal inference. J. Stat. Softw. 42, 1–28. 10.18637/jss.v042.i08

[B37] Swinscow T. Campbell M. (1997). Study design and choosing a statistical test. *Statistics at Square One* . London: BMJ Publishing Group.

[B38] Tasmanian Health Service (2019). Public sector Agreements. Available at: https://www.tic.tas.gov.au/public_sector_agreements (Accessed February 15, 2022).

[B39] Thomas R. Huntley A. L. Mann M. Huws D. Elwyn G. Paranjothy S. (2014). Pharmacist-led interventions to reduce unplanned admissions for older people: a systematic review and meta-analysis of randomised controlled trials. Age Ageing 43, 174–187. 10.1093/ageing/aft169 24196278

[B40] Tong E. Y. Mitra B. Yip G. Galbraith K. Dooley M. J. Group P. R. (2020). Multi-site evaluation of partnered pharmacist medication charting and in-hospital length of stay. Br. J. Clin. Pharmacol. 86, 285–290. 10.1111/bcp.14128 31631393 PMC7015749

[B41] Tong E. Y. Roman C. P. Smit D. V. Newnham H. Galbraith K. Dooley M. J. (2015). Partnered medication review and charting between the pharmacist and medical officer in the emergency short stay and general medicine unit. Aust. Emerg. Nurs. J. 18, 149–155. 10.1016/j.aenj.2015.03.002 26012888

