# Supplementary figures and images for: Clinical and economic impact of partnered pharmacist medication charting in the emergency department
Source: Front Pharmacol. 2023 Dec 8;14:1273657. doi: 10.3389/fphar.2023.1273657 (PMC10748591; doi:10.3389/fphar.2023.1273657)

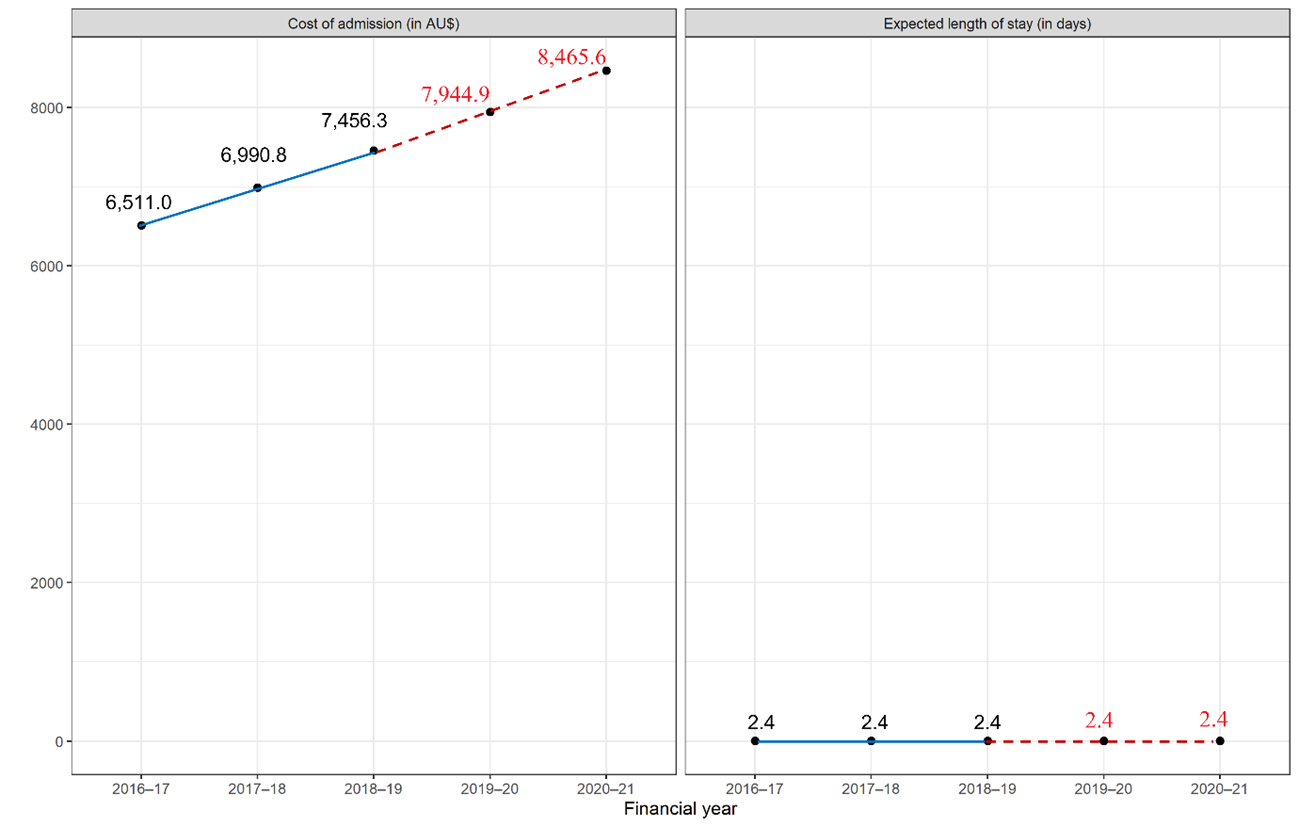

Supplement: Supplementary file 1 [file Image1.tif]
